# Supplementary material for: Whole genome sequencing characterization of Slovenian carbapenem-resistant Klebsiella pneumoniae, including OXA-48 and NDM-1 producing outbreak isolates
Source: PLoS One. 2020 Apr 13;15(4):e0231503. doi: 10.1371/journal.pone.0231503 (PMC7153892; doi:10.1371/journal.pone.0231503)
Supplement: S1 File — Six isolates were analysed using ONT GridIon. Four sequenced isolates were part of a confirmed outbreak: two belonged to the main cluster of ST437, the other two were selected as the only representatives of ST147 in order to confirm transmission of a plasmid with the blaOXA-48 resistance gene among different clones in the outbreak. One isolate of ST437 was previously classified as unrelated to the outbreak according to epidemiological data but clustered together with the outbreak isolates; therefore, we wanted to confirm it as part of the outbreak. The sixth isolate was sequenced because we detected blaLEN in K. pneumoniae belonging to ST258 and we wanted to confirm the presence of that gene. (DOC) [file pone.0231503.s003.doc]

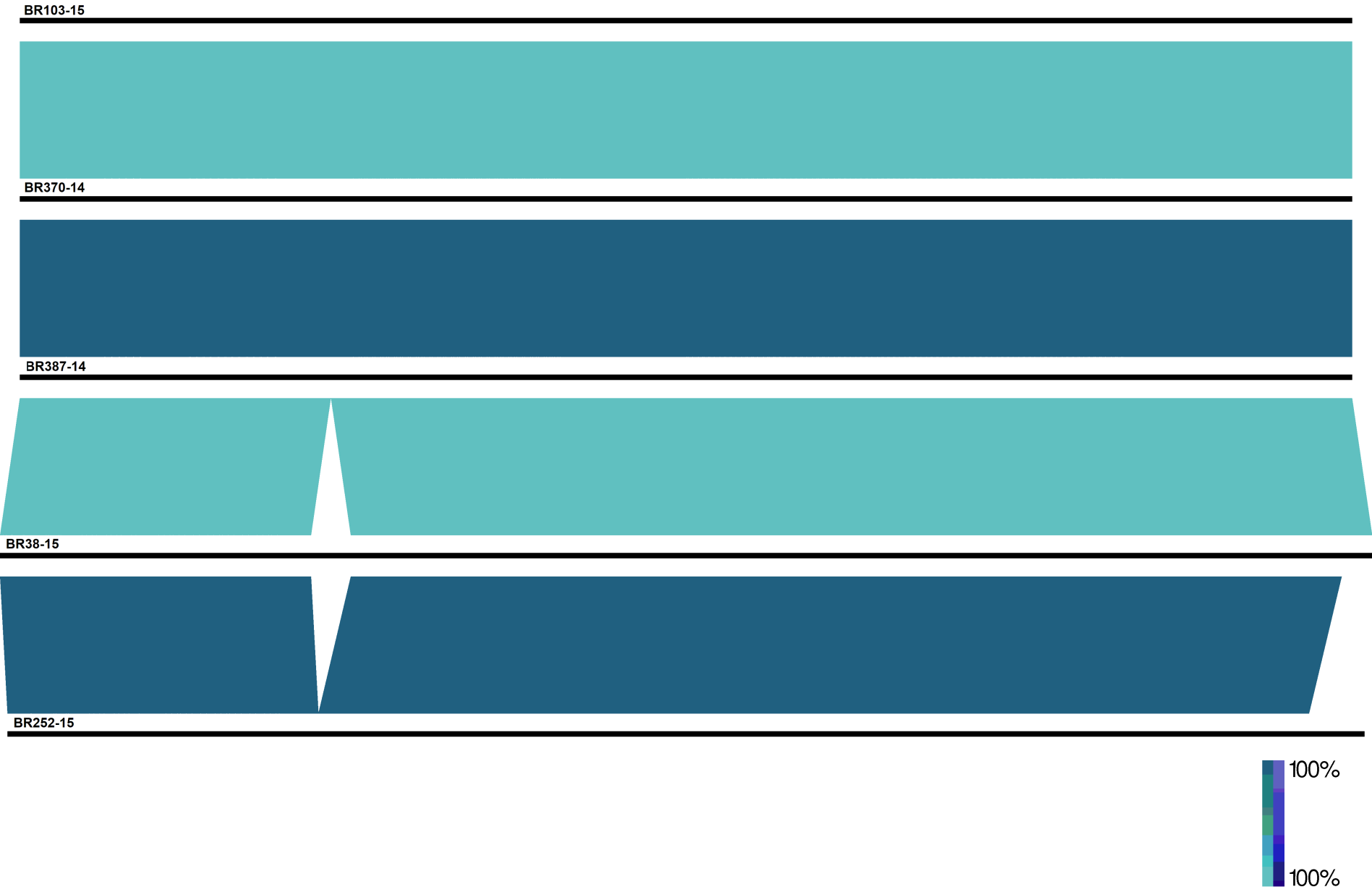


**Figure S1.**

EasyFig-generated schematic representation of *bla*OXA-48 encoding plasmids detected with long-read whole genome sequencing. Black lines represent plasmids from five *K. pneumoniae* isolates positive for *bla*OXA-48 in PCR and short-read whole-genome sequencing. Coloured bars represent shared parts of genome between plasmids. An insertion was detected in isolate BR38 (white triangular insert in the bottom two coloured bars).
