# Supplementary figures and images for: Whole genome sequencing characterization of Slovenian carbapenem-resistant Klebsiella pneumoniae, including OXA-48 and NDM-1 producing outbreak isolates
Source: PLoS One. 2020 Apr 13;15(4):e0231503. doi: 10.1371/journal.pone.0231503 (PMC7153892; doi:10.1371/journal.pone.0231503)

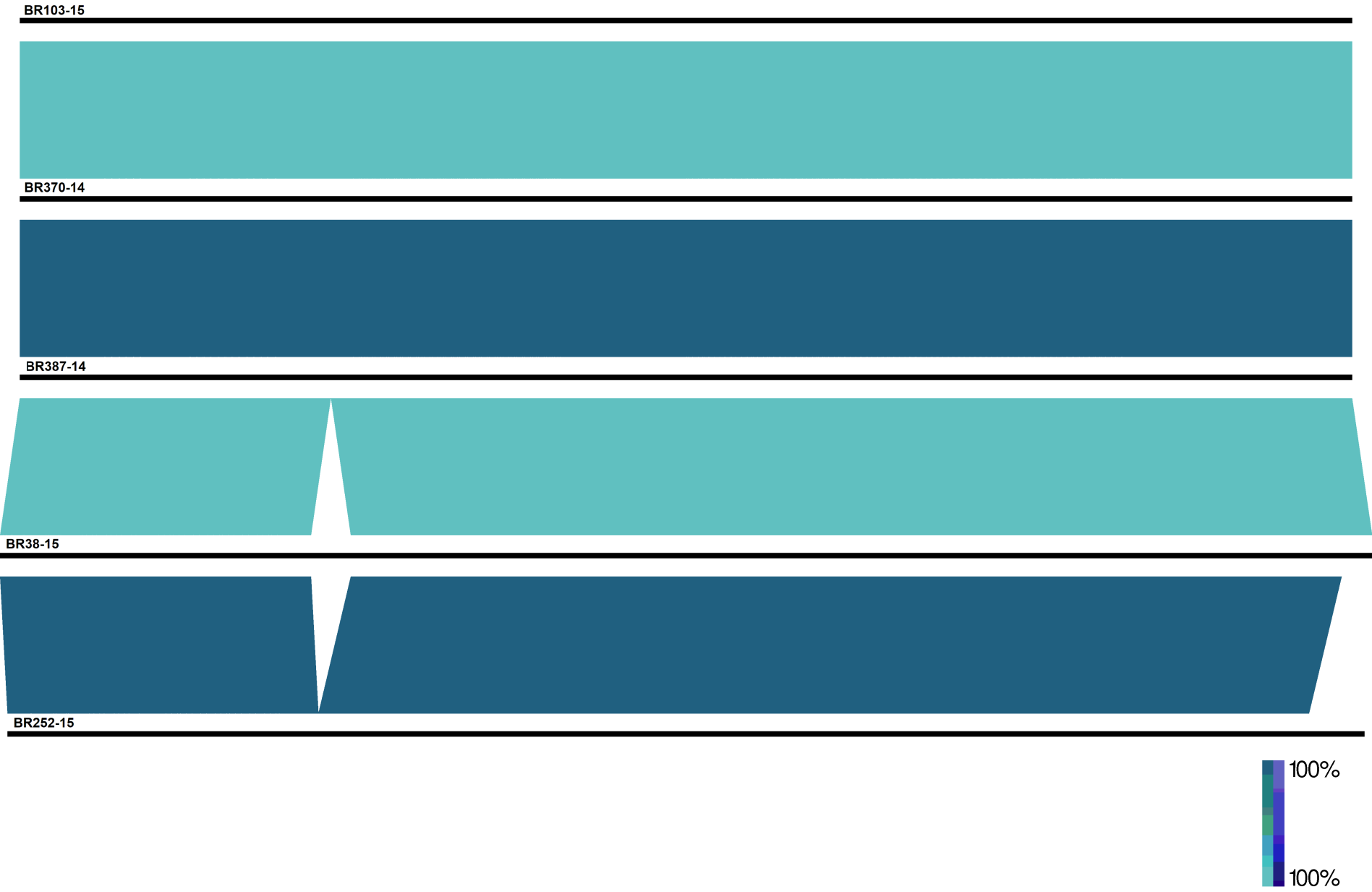

Supplement: S1 Fig — Black lines represent plasmids from five K. pneumoniae isolates positive for blaOXA-48 in PCR and short-read whole-genome sequencing. Coloured bars represent shared parts of genome between plasmids. An insertion was detected in isolate BR38 (white triangular insert in the bottom two coloured bars). (TIF) [file pone.0231503.s004.tif]
