# Supplementary material for: Whole genome sequencing characterization of Slovenian carbapenem-resistant Klebsiella pneumoniae, including OXA-48 and NDM-1 producing outbreak isolates
Source: PLoS One. 2020 Apr 13;15(4):e0231503. doi: 10.1371/journal.pone.0231503 (PMC7153892; doi:10.1371/journal.pone.0231503)
Supplement: Attachment — Submitted filename: Response to Reviewers.docx [file pone.0231503.s005.docx]

**Line 120- Quality control and filtering of sequences have been performed?? Authors should make ensure that the sequences have enough quality to do the subsequent bioinformatics analysis. In the same way, they should include a quality assembly report (QUAST report or similar with N50, L50, number of contigs, length of genomes, etc.) that allows readers to know the quality of the draft genomes obtained after the assembly.**

We have included the information in the revised version of the article. Please see lines 121-127 and S1 Table.

**Line 208-210- A reference plasmid has not been represented along with plasmids identified during this study. To understand the epidemiological relevance of these OXA-48-encoding plasmids and their transmission in this country, they should be compared with the reference plasmids more closely related (for example the worldwide disseminated pOXA-48a described by Poirel in 2012). Alike, plasmid representation should be annotated (the genetic environment of carbapenemase genes plays an important role in the dissemination of these genes and should be represented in the figure 2).**

Our five ONT sequenced pOXA-48 positive isolates are now annotated and compared to the reference plasmid described by Poirel et al. (2012). Please see lines 149-155, 224-227, line 321 and Figure 2.

**Line 211- Plasmids with OXA-48 and NDM-1 were different? Information about this strain and both plasmids is not included in the study. Has the OXA-48-encoding plasmid been compared with the other 5 sequenced plasmids? These data must be included in the study.**

Thank you for your comment. In the strain with both, OXA-48 and NDM-1 plasmid (BR252-15), OXA-48 plasmid was compared to four other sequenced plasmids and the comparison is seen in the Figure 2. In the 6^th^ ONT sequenced isolate, as expected, we did not detect OXA-48 or NDM-1 plasmid. Please see lines 102-104 and also lines 229-232.

**Line 236- In the discussion, authors say that the combination blaOXA-48, blaNDM-1 and blaCTX-M-15 of genes is firstly detected in the ST437 clone. CTX-M enzymes has not been previously mentioned in the results section. If authors consider that this is an important finding, they should be included the result in the paragraph “Detection of beta-lactamase genes in WGS data” (not only in the table of supplementary material). The same in lines 238-240.**

Thank you for your comment. The information was added, please see lines 178-180.

**Line 262- Alike, blaLEN results have been firstly commented in the discussion and non-mention has been previously made in the Results.**

The information was added, please see lines 178-180.

**Lines 295-301-** **These results should be better explained in the results section and then express a more general idea in the discussion.**

Changes were made to the results and discussion sections regarding the plasmids. Please see lines 220-222 and also lines 317-324.

**Line 58 and 224 - Authors comment that most frequent clones in Europe are ST11, ST15, ST101 and…ST285. With the latter the author must refer to clone ST258.**

The mistake was corrected.

**Line 59 - Authors should use “present simple” form throughout all the sentence (…ST101 are associated with carbapenemase….ST17 usually carry extended spectrum…)**

The sentence was corrected.

**Line 108 – Which are the breakpoints used for the antimicrobial susceptibility study? (EUCAST-2019 or 2018 or 2017??).**

For AST with disk diffusion contemporary EUCAST guidelines were used (with the ending of the isolate name denoting the year of isolation). The information was added to lines 109-110.

**Lines 116-118 – Kits for library preparations are indicated in this section, but not the sequencing platform used (Illumina HiSeq? NovaSeq?)**

The information regarding sequencing platform was added. Please see lines 120-121.

Please note: There was a minor change to S1_File_Methods. Figure S2, which was not referred to in the manuscript and not intended for publication, was accidentally included in the previous submitted version. It has been removed from the current submission.
